# Production of (R)-3-hydroxybutyric acid by *Burkholderia cepacia* from wood extract hydrolysates

**DOI:** 10.1186/s13568-014-0028-9

**Published:** 2014-03-18

**Authors:** Yuanzhen Wang, Shijie Liu

**Affiliations:** 1 Department of Paper and Bioprocess Engineering, SUNY College of Environmental Science and Forestry, 1 Forestry Drive, Syracuse 13210, NY, USA

**Keywords:** (R)-3-hydroxybutyric acid, Burkholderia cepacia, Wood extract hydrolysate

## Abstract

(R)-hydroxyalkanoic acids (R-HAs) are valuable building blocks for the synthesis of fine chemicals and biopolymers because of the chiral center and the two active functional groups. Hydroxyalkanoic acids fermentation can revolutionize the polyhydroxyalkanoic acids (PHA) production by increasing efficiency and enhancing product utility. Modifying the fermentation conditions that promotes the *in vivo* depolymerization and secretion to fermentation broth in wild type bacteria is a novel and promising approach to produce R-HAs. Wood extract hydrolysate (WEH) was found to be a suitable substrate for R-3-hydroxybutyric acid (R-3-HB) production by *Burkholderia cepacia*. Using *Paulownia elongate* WEH as a feedstock, the R-3-HB concentration in fermentation broth reached as high as 14.2 g/L after 3 days of batch fermentation and the highest concentration of 16.8 g/L was obtained at day 9. Further investigation indicated that the composition of culture medium contributed to the enhanced R-3-HB production.

## Introduction

(R)-hydroxyalkanoic acids (R-HAs) are valuable chiral building blocks for synthesis of fine chemicals and pharmaceuticals, such as antibiotics, amino acids, vitamins, food supplements, cosmetics and many others (Chen and Wu [2005]; Lee et al. [1999]; Ren et al. [2010]). In addition, R-HAs can be used as precursors to produce homopolymers and “tailor-made” copolymers as well as to overcome the challenge of producing high molecular weight polymers through controlled *in vitro* polymerization (Ren et al. [2010]). The R-HAs contain at least one chiral center and two active functional groups: a hydroxyl group and a carboxyl group (Lee and Park [2009]). HAs being enantiomerically pure is important for their applications. For example, a chiral pharmaceutical possesses advantages including improved safety, enhanced efficiency, lower dosage and reduced side effects compared to traditionally synthesized compounds (Chen and Wu [2005]; Ren et al. [2010]).

Polyhydroxyalkanoates (PHAs) are a family of polyesters commonly known to be synthesized by bacteria as intracellular carbon and energy storage compounds (Wang and Liu [2013]). PHAs from microbial sources are all composed of chiral (R)-hydroxyalkanoic acids (Chen and Wu [2005]; Lee and Park [2009]; Lee et al. [1999]). To date, approximately 150 R-HA monomers have been identified (Steinbüchel and Lütke-Eversloh [2003]). Industrial production of PHA has been based on the intracellular synthesis, which is different from polylactic acid (PLA) production (Buyondo and Liu [2011]). Lactic acid is produced as an extracellular product, and then lactic acid is chemically polymerized to PLA. The polymer properties can be adjusted to some degree by adjusting the degree of polymerization. Apart from limited utility and/or narrow distribution of properties, intracellular PHA incurs significant cost in extraction, separation and purification (Chen [2009]; Choi and Lee [1999]). It is therefore desirable to examine the potential for the extracellular production/modification of PHAs to expand their utility.

Currently, methods for production of R-HAs include chemical synthesis, biocatalysis, chemical or enzymatic degradation of biologically synthesized Polyhydroxyalkanoates (PHAs), *in vivo* depolymerization of PHAs, and biotechnological production by metabolic pathway engineering (Chen and Wu [2005]; Ren et al. [2010]). Direct chemical synthesis of R-HAs has many limitations such as the requirement of pure substrate and expensive catalysts (Ren et al. [2005]; Ren et al. [2010]). Using microorganisms as biocatalysts for R-HA production often gives low yields and enantiomeric excesses (Ren et al. [2005]). *In vivo* depolymerization of PHAs is disadvantageous due to the low efficiency (de Roo et al. [2002]; Kawata et al. [2012]). *In vivo* depolymerization of PHA requires the biomass with PHA accumulated to be harvested from fermentation broth first, and then PHA degradation is enhanced by modifying the pH or temperature (Lee et al. [1999]; Ren et al. [2005]). Metabolic pathway engineering has been employed to produce R-HAs by over-expression of depolymerase and/or under-expression of synthase or dehydrogenase, but it is still at a stage in need of practical use (Chung et al. [2009]; Park et al. [2004]; Romano et al. [2005]; Sandoval et al. [2005]).

Production of R-HAs by wild-type bacteria fermentation through modifying the culture conditions is a very promising and innovative approach (Kawata et al. [2012]; Ren et al. [2010]). R-HA is then an extracellular product, which is more efficient because it can be harvested directly from fermentation broth. To date, there is very little research exploring the direct fermentation method for R-HA production. Applying successive aerobic and microaerobic culture condition has been reported to increase the extracellular accumulation of R-HAs by *Halomonas* sp. KM-1 in 200 mL Erlenmeyer flasks culture (Kawata et al. [2012]).

In our previous publications, the production of polyhydroxybutyric acids (PHB) by *Burkholderia cepacia* was reported using wood extract hydrolysate (Keenan et al. [2004]; Liu [2012]; Wang et al. [2012]). In this paper, we investigated a novel approach for the production of (R)-3-hydroxybutyric acid (R-3-HB) by *Burkholderia cepacia* fermentation through alteration of culture conditions and using wood extract hydrolysate as the fermentation substrate. To the best of our knowledge, it is the first time reporting utilizing wild type *Burkholderia cepacia* to directly produce R-3-HB.

## Materials and methods

### Preparation of *Acer saccharum* WEH

Hot water extraction on *Acer saccharum* woodchips was carried out at 160°C for 2 h. The resulting suspension was then subjected to a membrane filtration to remove the suspended solids and to concentrate the wood extract. Dilute acid hydrolysis of the wood extract was then performed with sulfuric acid (1.5 wt%) at 120°C for 30 min. Wood extract hydrolysate was centrifuged to remove solid deposits before neutralized by calcium hydroxide and again centrifuged to remove the precipitate (CEPA centrifuge Z81G). Ten times dilution of the hydrolysate before nano-filtration was performed and repeated twice in order to remove and to recover fermentation inhibiting compounds such as acetic acid, furfural, and 5-hydroxymethylfurfural (Shupe and Liu [2012b]; Sun and Liu [2012]).

### Preparation of *Paulownia elongata* WEH

Four year old *Paulownia elongata* wood logs were obtained from Fort Valley State University, Fort Valley in Georgia and were chipped with bark-on and screened to a uniform size of 2.5 × 2.0 × 0.5 cm^3^. A 1.84 m^3^ digester with external steam heating system was used to carry out the hot-water extraction. Water to solid ratio was 7.69:1. The extraction was conducted nominally at 160˚C for 2 h (Yan et al. [2013]). The extraction liquor was devoid of solids via ultra-filtration and then concentrated via nano-filtration. Dilute acid hydrolysis of the concentrated wood extract was then performed with mixed acids: hydrochloric acid (0.1 wt%), nitric acid (0.1 wt%), and sulfuric acid (0.8 wt%) at 121°C for 60 min. The hydrolysate was devoid of solids through paper filtration before neutralization with calcium hydroxide and sodium hydroxide. The neutralized wood extract hydrolysate was then filtered to remove solids with filter paper. Ten times dilution of the hydrolysate followed by nano-filtration was performed and repeated in order to remove and to recover fermentation inhibiting compounds such as acetic acid, furfural, and 5-hydroxymethylfurfural (Shupe and Liu [2012b]; Sun and Liu [2012]).

### Batch fermentation

*Bukerholderia cepacia* ATCC 17795 was purchased from American Type Culture Collection. The batch fermentations of *B. cepacia* utilizing untreated 50 g/L xylose, 50 g/L glucose, 50% sugar maple WEH, and 50% *Paulownia elongata* WEH were performed in a 1.0 L bioreactor (Bioflo 110, Newbrunswick, USA). For the experiments of simulating *Paulownia elongata* WEH by adding extra nitrate and chloride ions to xylose solution, calcium nitrate and calcium chloride was added at the same amount as introduced by dilute acid hydrolysis.

The working volume was 500 mL, contained 50 mg CaCl_2_, 30 mg NH_4_ Fe(III) citrate, 100 mg MgSO_4_, and 0.07% levulinic acid (Bertrand et al. [1990]; Keenan et al. [2004]). Na_2_HPO_4_ and Na_2_PO_4_ were added with a weight ratio of 1:1.35 to achieve a final 0.02 mol/L phosphate ion concentration. The nitrogen source is limited by reducing the concentration of (NH_4_)_2_SO_4_ to 1.5 g/L (Keenan et al. [2004]). The pH was maintained at 7.0 by adding 5 M NaOH. Excessive foaming was detected by a foam probe and defoaming was carried out by addition of silicone based antifoam.

The biomass is harvested from fermentation broth by centrifugation at 4000 × g for 10 min followed by freeze drying and then weighted to obtain the dried weight of the biomass. The freeze dried biomass was suspended into 50 mL chloroform (5% wt/vol) and incubated in 250 mL sealed glass flasks at 60°C for 24 h. Chloroform solution was obtained by filtration with whatman #1 filter paper, and then methanol was added to the PHB solution at ration of 5:1 (vol/vol). PHB was therefore precipitated and harvested by centrifugation at 4000 × g for 20 min. Polymer films were prepared by re-dissolving polymer with chloroform in 10 cm glass Petri dishes followed by evaporating chloroform in a fume hood for overnight. The PHB polymer was then weighted to obtain the PHB yield.

### Analytical methods

Sugar concentrations in WEH were analyzed using nuclear magnetic resonance (NMR) spectroscopy (AVANCE, Bruker BioSpin co.) operated at 600 MHz. A modified two dimensional (2D) heteronuclear single quantum coherence (HSQC) method was used to determine the sugar concentration (Shupe et al. [2012]; Shupe and Liu [2012a]).

Characterization and quantification of (R)-3-hydrobutyric acid was obtained by analyzing ^1^H nuclear magnetic resonance spectroscopy (AVANCE 600, Bruker BioSpin co.) operated at 600 MHz. The concentration of R-3-HB was calculated by integrating the peak area at 1.12 ppm corresponding to the peak area of internal standard trimethylamine (TMA) with known concentration of 2.28 g/L.

(1) $$\frac{P_{\mathrm{HB}}}{P_{\mathrm{TMA}}}=\frac{M_{\mathrm{HB}}\times 3}{M_{\mathrm{TMA}}\times 9}$$

Where P_HB_ and P_TMA_ are the integrated peak area of R-3-HB at 1.12 ppm and TMA at 2.90 ppm, and M_HB_ and M_TMA_ are mole concentrations of R-3-HB and TMA respectively.

## Results

### Batch fermentation using wood extract hydrolysates (WEH)

**Figure 1:**
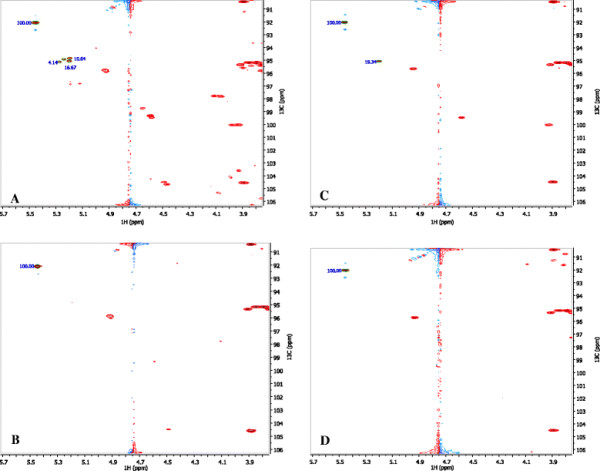
**2D HSQC spectrum for wood exact hydrolysate (WEH) (A:** ***Paulownia elongate*** **WEH before** ***B.cepacia*** **fermentation; B:** ***Paulownia elongate*** **WEH after 11 days** ***B.cepacia*** **fermentation; C:** ***Acer saccharum*** **WEH before** ***B.cepacia*** **fermentation; D:** ***Acer saccharum*** **WEH after 10 days** ***B.cepacia*** **fermentation).**

NMR 2D HSQC spectrums with chemical shifts ranging from 4.1 to 6.0 ppm for ^1^H (f2) and 91 to 106 ppm for ^13^C (f1) was obtained for sugar concentration determination in wood extract hydrolysates (WEH) as shown in Figure 1. The concentrations of monosaccharides were calculated using the method reported previously (Shupe et al. [2012]). First the peaks of each monosaccharaide and oligomers were identified in the 2D-HSQC spectrum and then their concentrations were calculated using the normalized peak areas using glucosamine (5.49, 92.10) as the internal standard. Approximate peak locations of the main components in WEH are: α-xylose (5.20, 95.06), α-glucose (5.23, 94.91), α-galactose (5.27, 95.12).

In *Paulownia elongate* WEH, there are 12.3 g/L galactose, 19.5 g/L glucose, and 39.5 g/L xylose before *B. cepacia* batch fermentation (Figure 1A). Xylose is the most abundant monosaccharide in *Acer saccharum* WEH, which has a concentration of 47.2 g/L before batch fermentation (Figure 1C). After 10 and 11 days of *B. cepacia* fermentation, almost all of the monosaccharides are consumed (Figure 1B and D). Xylo-oligomers (4.47, 104.3) were also detected in *Paulownia elongate* WEH as shown in Figure 1A. However, these oligomers were consumed to a large extent after 11 days of fermentation (Figure 1C), which indicated the ability of *B. cepacia* to utilize oligosaccharides.

### Characterization and quantification of R-3-HB

**Figure 2:**
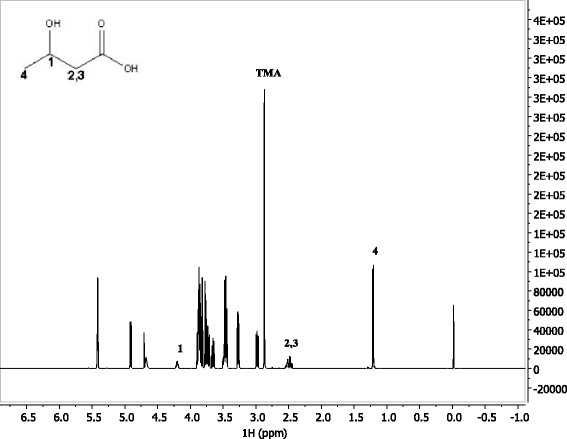
Proton NMR spectrum of reagent 3-HB.

**Figure 3:**
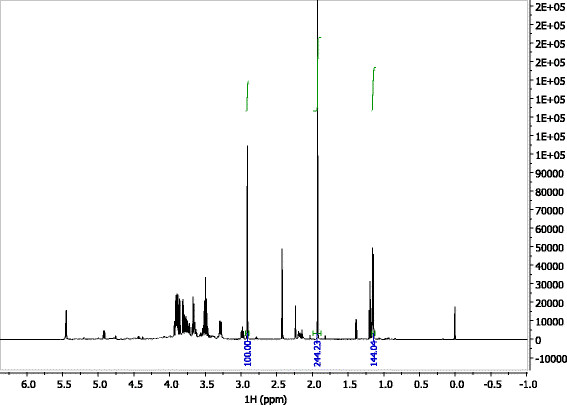
**Proton spectrum of** ***B. cepacia*** **fermentation broth after 8 days of fermentation using** ***Paulownia elongate*** **WEH as substrate.**

The proton NMR spectrum of a regent 3-HB in water solution is shown in Figure 2. The resonance peaks at 1.2 ppm, 2.5 ppm, and 4.2 ppm correspond to the labeled protons on the 3-HB molecule. These resonance peaks of 3-HB were also identified on the proton spectrum of *B. cepacia* fermentation broth (Figure 3). Because the peaks at 2.5 ppm and 4.2 ppm overlap with resonance peaks of other compounds in the fermentation broth, we only integrate peak area of the double peak located at 1.2 ppm to quantify the concentration of 3-HB.

**Figure 4:**
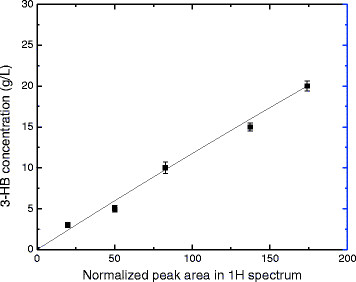
Standard curve of normalized peak area of 3-HB in proton spectrum corresponding to its concentration.

**Figure 5:**
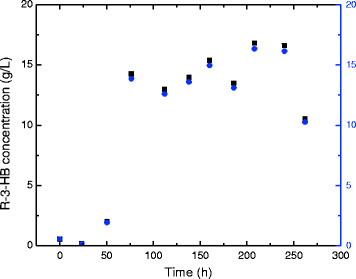
3-HB concentrations in the fermentation broth determined by NMR readings (■) and standard curve (blue circle symbol).

A linear regression line (y = 0.1134x, R^2^ = 0.9937) was obtained using the regent 3-HB standard solutions (Figure 4). The normalized peak area of 3-HB at 1.2 ppm based on internal standard (trimethylamine, TMA) showed a positive linear correlation with the concentration of 3-HB at the range of 0 g/L to 20 g/L. To ascertain the quantitative nature of the proton NMR, Figure 5 shows a comparison of the concentration as determined from NMR reading and those of standard solutions. One can observe from Figure 5 that the concentration of R-3-HB determined by NMR agrees very well with the actual concentration in the solution.

### R-3-HB production from WEH

From the proton spectrum of *B. cepacia* fermentation broth using *Paulownia elongate* WEH as a substrate (Figure 3), right shifting of resonance peaks of 3-HB is observed. For example, the CH resonance peak located at 1.20 ppm for the regent 3-HB, but it shifted slightly right to 1.12 ppm for the 3-HB produced in the fermentation broth. This resonance peak shifting is most probably resulted from neutralization of acids by sodium hydroxide. Sodium hydroxide was dosed into the medium automatically and consistently in order to maintain the pH at 7.0 in the fermentation.

In addition to 3-HB, acetic acid which has a resonance peak at 1.90 ppm, was also identified in the fermentation broth from the proton spectrum (Figure 3). The accumulation of acetic acid started at day 3 and reached the peak concentration of 16.6 g/L at day 11 (240 h), and then decreased to 12.4 g/L at day 12 (262 h). The metabolism of acetic acid production by *B. cepacia* is beyond the research scope of this paper, and will be further investigated.

**Figure 6:**
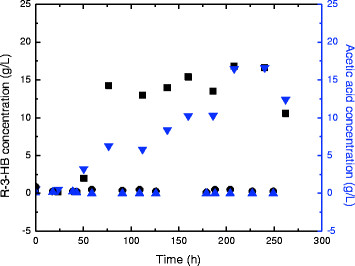
**R-3-HB production during batch fermentation of B. cepacia using 50%** ***Acer saccharum*** **WEH or 50%** ***Paulownia elongate*** **WEH as substrate (R-3-HB in** ***Acer saccharum*** **WEH: •; R-3-HB in** ***Paulownia elongate*** **WEH: ■; Acetic acid in** ***Acer saccharum*** **WEH: blue up pointing triangle symbol; Acetic acid in** ***Paulownia elongate*** **WEH:blue down pointing triangle symbol).**

Production of R-3-HB by *B. cepacia* using *Acer saccharum* WEH and *Paulownia elongate* WEH is significantly different (Figure 6). Almost no R-3-HB can be detected in the fermentation broth using *Acer saccharum* WEH during the whole process. On the other hand, production of R-3-HB was detected in the medium using *Paulownia elongate* WEH during the third day of fermentation. The R-3-HB production started at day 3 (50 h), and then a significant increase to 14.2 g/L was observed at day 4 (76 h). The highest HB centration reached was 16.8 g/L at day 9 (208 h), which was then followed by a decreasing trend to 10.5 g/L at day 12 (262 h).

### Investigation of R-3-HB production using simulated WEH

Compared to *Acer saccharum* WEH, *Paulownia elongate* WEH has much more six carbon monosaccharides, mainly glucose and galactose, as well as a significant amount of oligosaccharides. Furthermore, the dilute acid hydrolysis process introduced extra chloride and nitrate ions into the *Paulownia elongate* WEH.

To investigate the metabolism that triggered the production of R-3-HB from *Paulownia elongate* WEH, we designed a series of batch fermentations. 50 g/L glucose solution, 50 g/L xylose solution, 50 g/L xylose solution with extra nitrate ion, and 50 g/L xylose solution with extra nitrate and chloride ions were all used to simulate the *Paulownia elongate* WEH as fermentation substrate.

**Figure 7:**
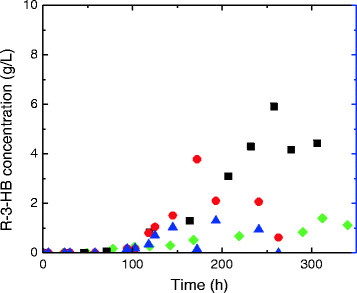
R-3-HB production during batch fermentation using 50 g/L glucose or 50 g/L xylose as substrate (xylose: green diamond symbol; glucose: ■; xylose with extra nitrate and chloride ions red circle symbol; xylose with extra nitrate ion blue up pointing triangle symbol).

Results of fermentation using glucose solution and xylose solution indicated that glucose as the fermentation substrate favors the production of R-3-HB more than using xylose (Figure 7). Introduction of extra nitrate and chloride ions seems to have a positive influence on the extracellular accumulation of R-3-HB.

The maximum R-3-HB concentration achieved from simulated WEH was 5.9 g/L from glucose. Using xylose as the fermentation substrate, highest R-3-HB concentration achieved is 3.8 g/L with extra nitrate and chloride ions. However, the extracellular concentration of R-3-HB reached 16.8 g/L using *Paulownia elongate* WEH. Some other compounds in the *Paulownia elongate* WEH might also contribute to the high R-3-HB production.

### Biomass and PHB yield in *B. cepacia* fermentation

During the balanced growth phase, synthesis of PHA has been observed to occur simultaneously with cell growth and reproduction (Braunegg et al. [1998]; Lee et al. [1999]). However, the microbial PHA accumulation has been observed to increase significantly during imbalanced growth (Braunegg et al. [1998]; Sudesh et al. [2000]), such as nitrogen (Tappel et al. [2012]), phosphorus (de Roo et al. [2002]), oxygen (Lv et al. [2012]), magnesium (Hu et al. [2011]), or sulfate limited conditions with excess carbon (Braunegg et al. [1998]; Riis and Mai [1988]). PHA production has generally been understood as induced by lack of one or more key nutrient for growth.

**Figure 8:**
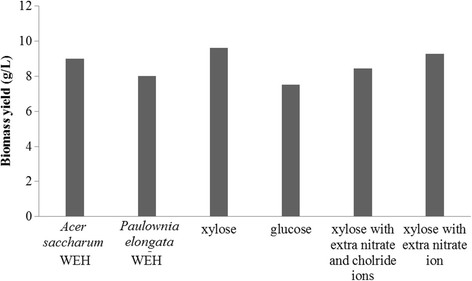
Biomass yield form batch fermentations.

**Figure 9:**
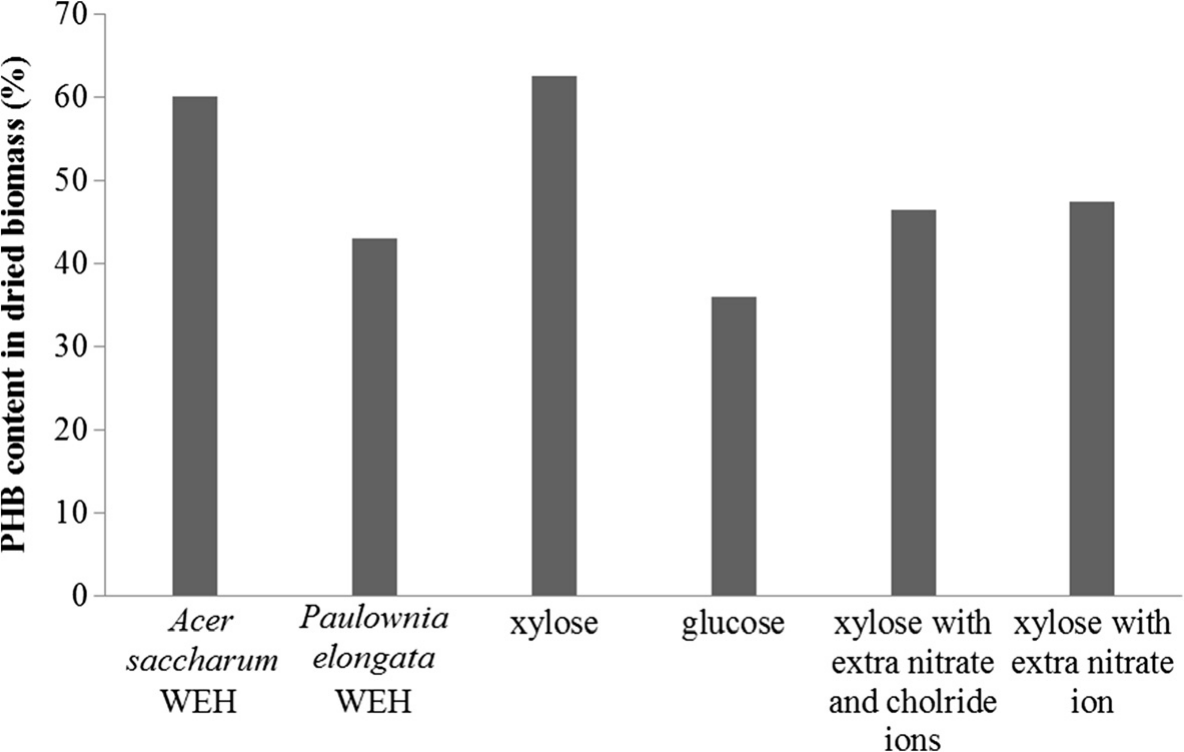
PHA content in dried biomass from batch fermentations.

**Figure 10:**
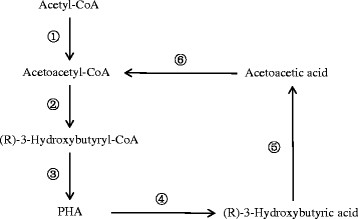
**Metabolic pathways of the biosynthesis and intracellular degradation of PHA.** ① PhaA, b-ketothiolase; ② PhaB, NADPH-dependent acetoacetyl-CoA reductase; ③ PhaC, PHA synthase; ④ PhaZ, PHA depolymerase; ⑤ (R)-3-Hdyroxybutyrate dehydrogenase; ⑥ Acetoacetyl-CoA synthetase.

In this study, nitrogen limited culture condition was adapted in the batch fermentations. The biomass yield and PHB content in the harvested biomass from six batch fermentations using 50% *Acer saccharum* WEH, 50% *Paulownia elongate* WEH, 50 g/L glucose, 50 g/L xylose, 50 g/L xylose with extra nitrate ion, and 50 g/L xylose with extra nitrate and chloride ions as substrate (Figures 8 and 9) respectively agreed with previous reported phenomenon that synthesis of PHA generally occur simultaneously with cell growth and reproduction (Braunegg et al. [1998]; Lee et al. [1999]). Another noteworthy phenomenon observed from these batch fermentations is that higher concentration of extracellular R-3-HB was detected from the fermentations with lower biomass and PHB yield, while less R-3-HB was produced from the fermentations with higher biomass and PHB yield, which confirms the mechanism proposed in Figure 10 that extracellular R-3-HB is generated by intracellular depolymerization of PHB followed by secretion. Using *Acer saccharum* WEH and xylose solution as substrate obtained the highest biomass and PHB yield, but produced negligible amount of R-3-HB monomers. On the other hand, high concentration of R-3-HB was produced from *Paulownia elongate* WEH with comparatively lower yield of biomass and polymers.

## Discussion

NMR has been successfully used to identify and quantify HAs (Lee et al. [1999]; [Seebach et al. 2003]). Furthermore, NMR can be used to identity and quantify HA along with other components in the fermentation broth, including monosaccharaides, oligomers, and other by-products. NMR samples can be run in minutes, yielding both proton and 2D-HSQC spectrums from a single sample, while eliminating difficulty in solvent selection and standard solutions. It is a well acknowledged quantitative analysis method in chemistry (Shupe et al. [2012]).

Currently, there are about 150 monomers that have been identified from the naturally produced PHAs (Steinbüchel and Valentin [1995]). It has been well acknowledged that all of these monomers are in the (R)-(−)-configuration if they have chiral center at the carbon connects to the hydroxyl group (Gangoiti et al. [2010]; Lee [1996]; Lee et al. [1999]; Liu et al. [2007]; Matsumoto et al. [2013]; Park et al. [2004]; Romano et al. [2005]; Ruth et al. [2007]; Steinbüchel and Valentin [1995]).

It has been demonstrated that in many wild type bacteria, the *in vivo* biosynthesis and degradation of PHA simultaneously take place (Wang et al. [2013]). A model of the cyclic metabolism of PHA is proposed and shown in Figure 10 (Braunegg et al. [1998]; Holmes [1985]; Sudesh et al. [2000]; Sudesh and Iwata [2008]). From the metabolic pathways we can conclude that it is essential to increase or maintain a high activity of PHA depolymerase while lowering the activity of (R)-3-Hydroxylbutyrate dehydrogenase in order to increase the production of R-HAs (Shiraki et al. [2006]; Tokiwa and Ugwu [2007]; Yuan et al. [2008]). There have been studies carried out on HA production through genetic engineering approach by overexpressing PHA depolymerase gene and/or repressing the (R)-3-Hydroxylbutyrate dehydrogenase gene (Holscher et al. [2010]; Lee and Lee [2003]; Shiraki et al. [2006]; Ugwu et al. [2008]).

Results of fermentations using six different medium showed that the concentration of R-3-HB in the fermentation broth increases at the expense of PHB yield, which confirms that R-3-HB is generated from the intracellular degradation of PHB. The mechanism of R-3-HB leakage into the fermentation broth would require further investigation. Biomass yield is higher in fermentation using xylose as carbon substrate compared to glucose, which agrees with the phenomena of higher biomass yield in *Acer saccharum* WEH than in *Paulownia elongate* WEH because there is more xylose in *Acer saccharum* WEH while *Paulownia elongate* WEH has significant amount of glucose.

Previous reports have indicated that controlling pH of the fermentation medium can shift metabolic pathway to enhance *in vivo* depolymerization of PHAs and thus favor R-HAs production (Lee et al. [1999]; Ren et al. [2005]). Introducing successive aeration conditions has been reported to increase the intracellular degradation of PHB to (R)-3-HB, which was then secreted into the culture broth (Kawata et al. [2012]). From this study, we could conclude that the composition of the fermentation substrate is also an important factor for R-HA production.

In good agreements with previous reports on HA production, it was found that the HA monomer secreted into the fermentation medium can be consumed by the cells again during fermentation (Lee et al. [1999]). Therefore, it would be a more efficient strategy to produce HAs by removing the HA monomers continuously during the fermentation (Yuan et al. [2008]).

In conclusion, production of R-HB directly into the fermentation broth by suitable fermentation conditions enhances the prospect of biodegradable polymer PHA. High concentration (16.8 g/L) of R-3-HB was produced by *B. cepacia* from *Paulownia elongate* WEH. The existence of oligosaccharides, six carbon monosaccharides such as glucose, as well as extra electrolytes such as nitrate and chloride ions in the fermentation medium has been observed to contribute to accumulation of R-3-HB in the fermentation supernatant. R-3-HB is generated from the intracellular depolymerization of storage compound PHB followed by secretion into the fermentation broth. This study launched an innovative approach to efficiently and economically produce R-HA.

## Competing interests

The authors declare that they have no competing interests.
